# Supplementary material for: Investigation of Potency and Safety of Live-Attenuated Peste des Petits Ruminant Virus Vaccine in Goats by Detection of Cellular and Humoral Immune Response
Source: Viruses. 2023 Jun 5;15(6):1325. doi: 10.3390/v15061325 (PMC10302441; doi:10.3390/v15061325)
Supplement: Supplementary file 1 [file viruses-15-01325-s001.zip › viruses-2373878-supplementary.pdf]

Figure S1a. ID rapid PPR antigen dipstick field test results of nasal swab. Negative (-) control band visible test band not, questionable ((+)) control band visible and barely visible test band, low positive (+) test band visible but not strong color as control band, moderate positive (++) test band same visible color as control band, high positive (+++) test band stronger color than control band.

Nasal swab 0 dpv

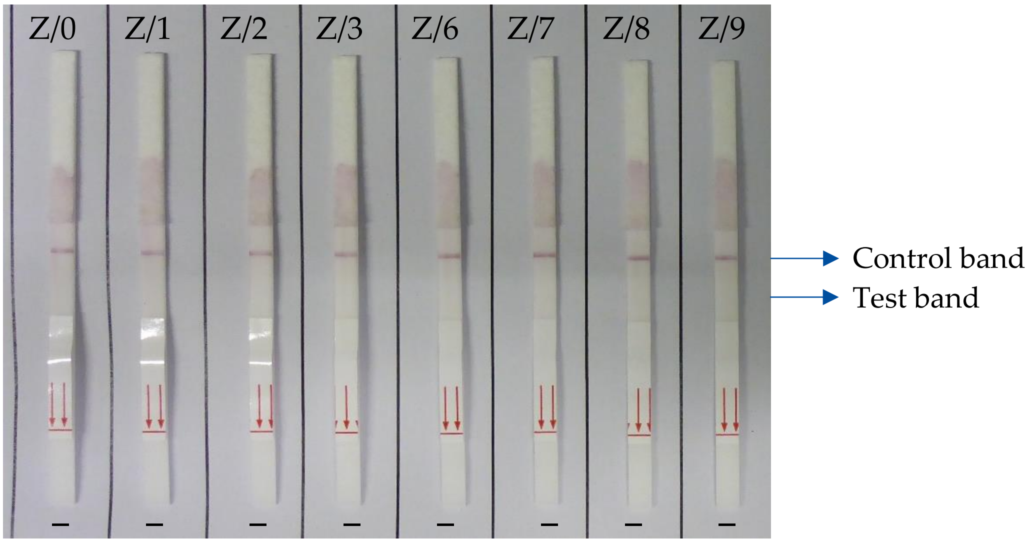

Nasal swab 3 dpv

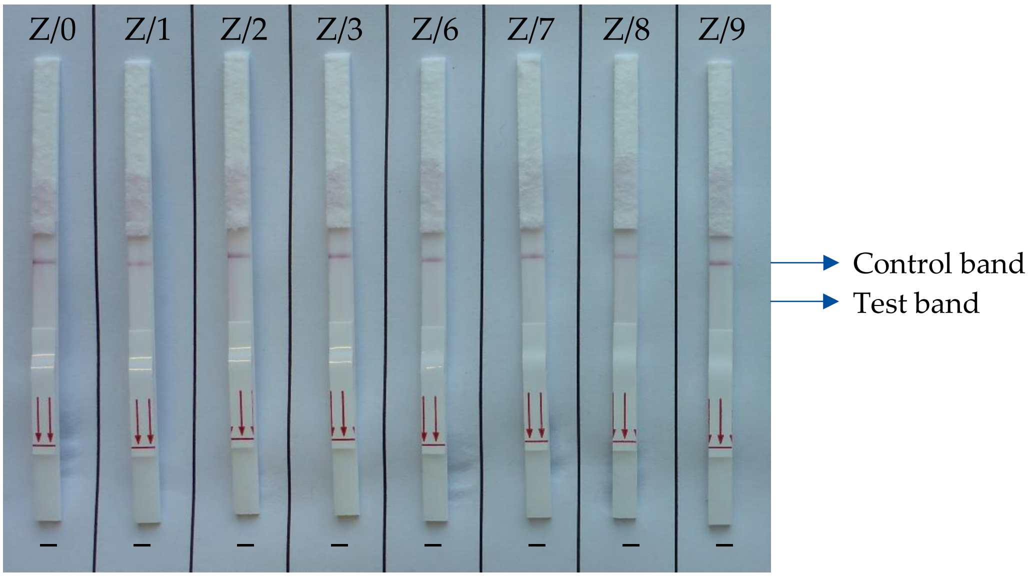

Nasal swab 5 dpv

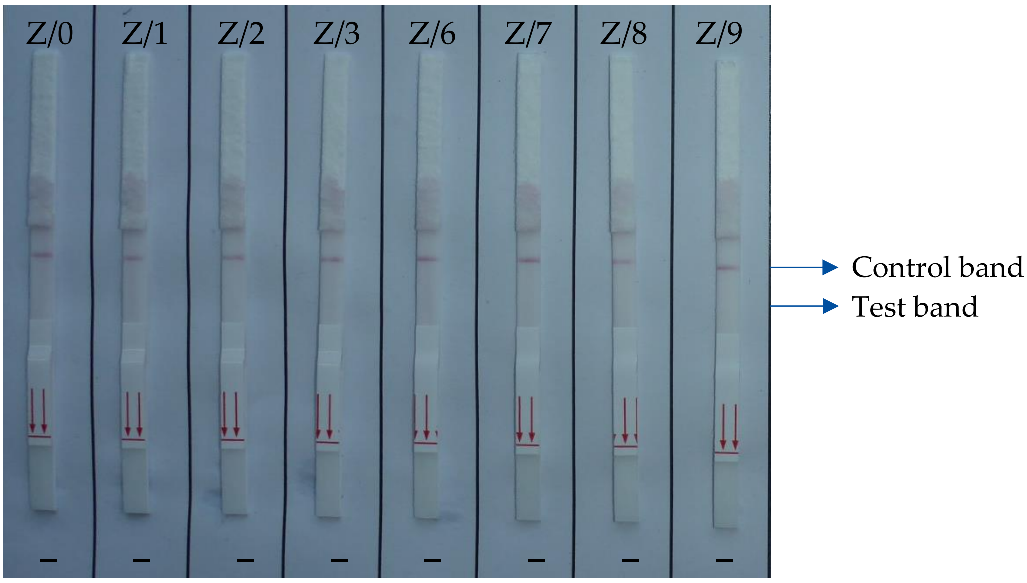

Nasal swab 7 dpv

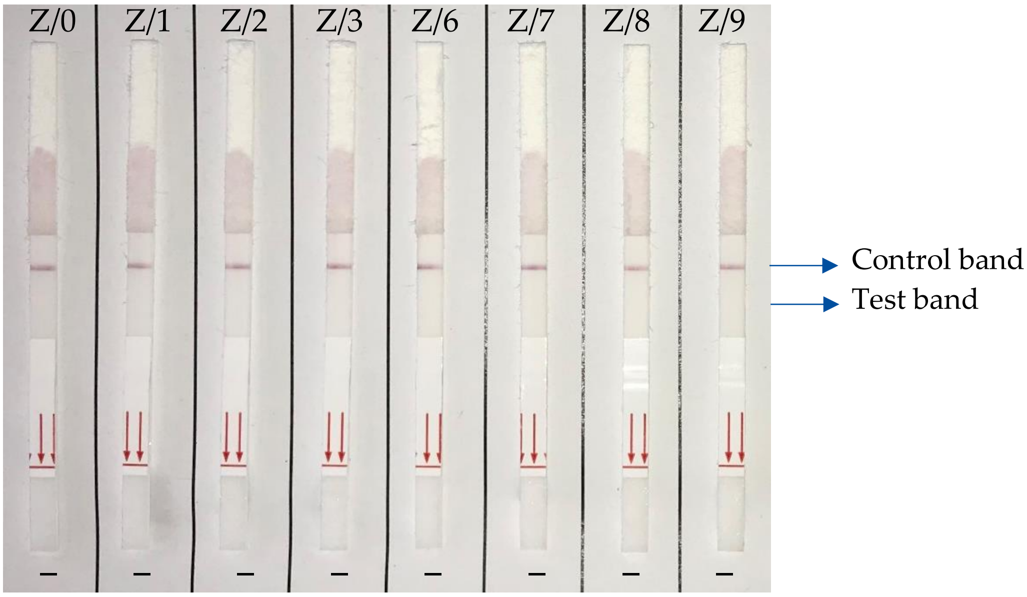

Nasal swab 10 dpv

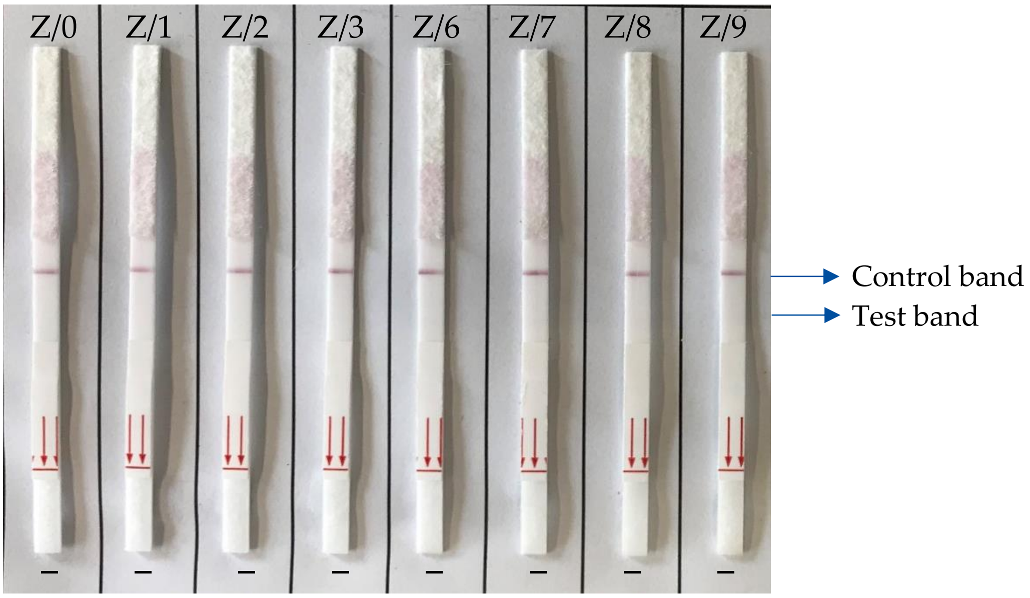

Nasal swab 12 dpv

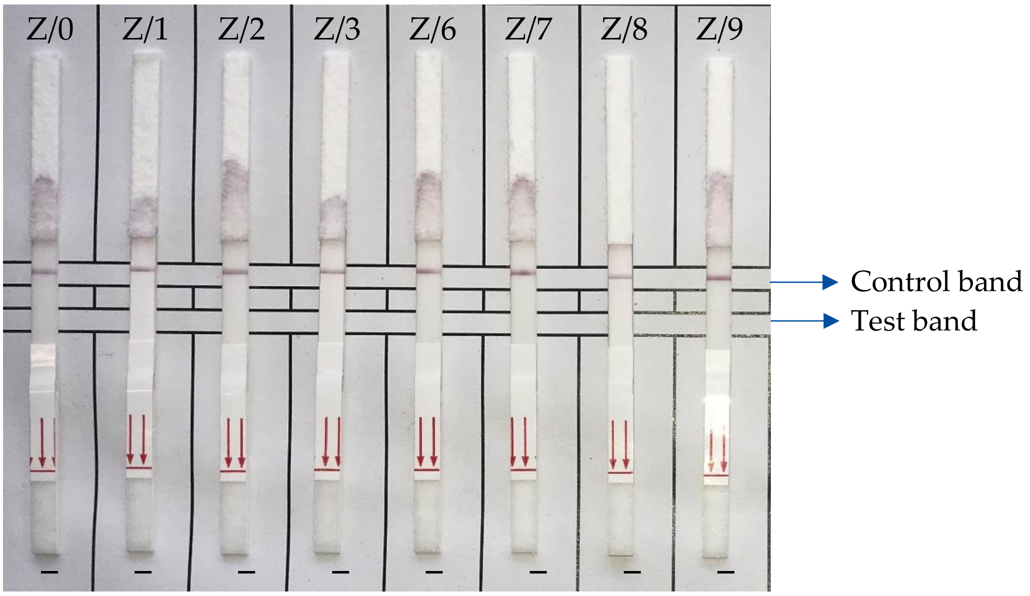

Nasal swab 14 dpv

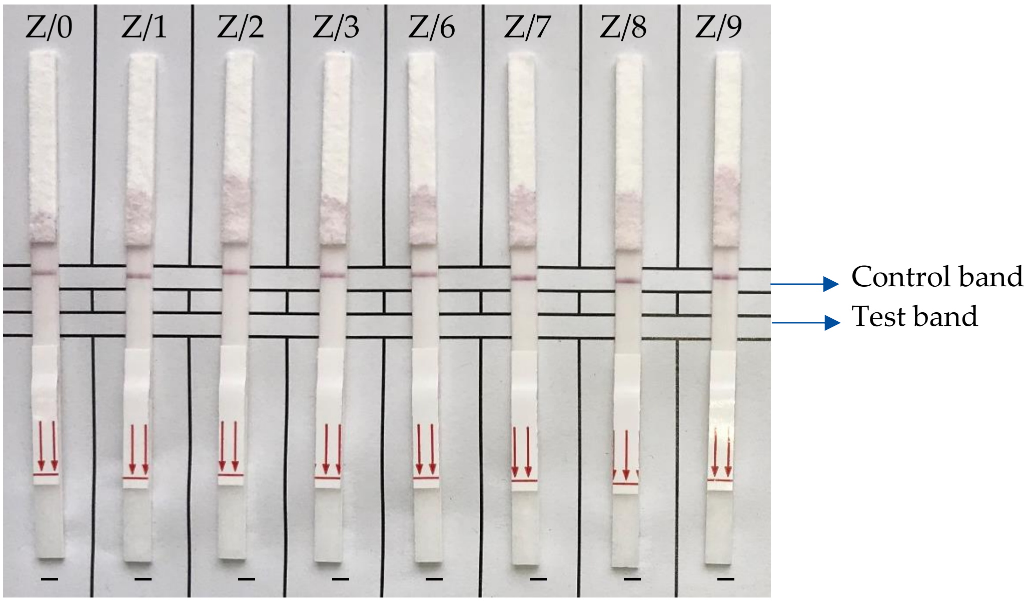

Nasal swab 17 dpv

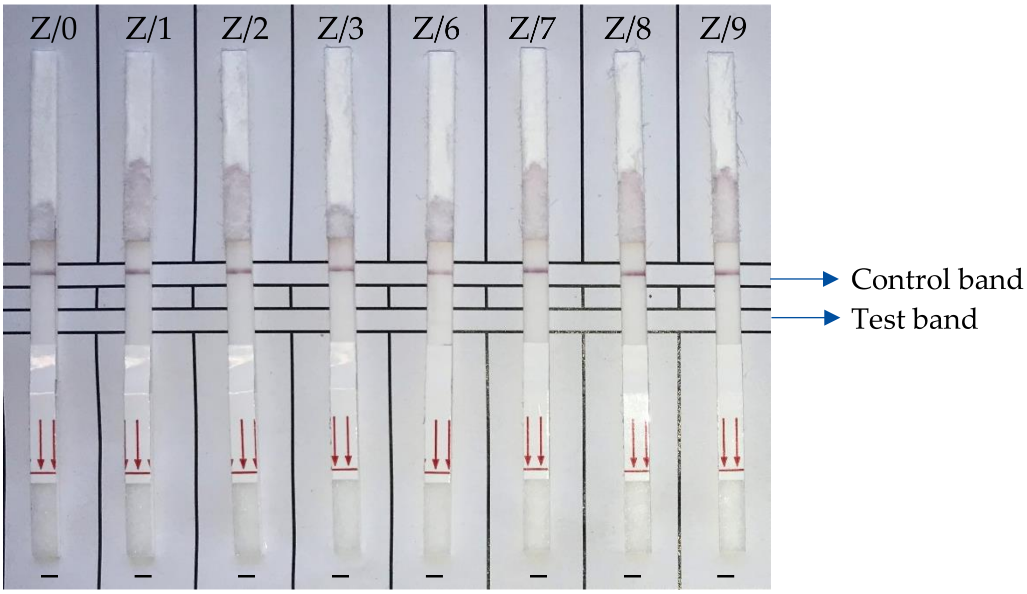

Nasal swab 21 dpv

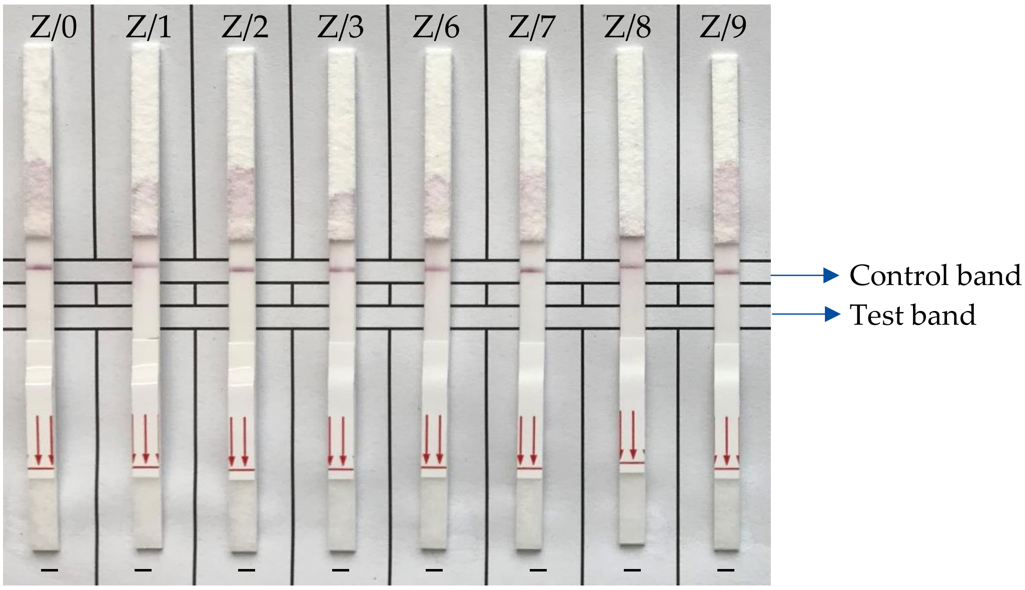

Nasal swab 24 dpv

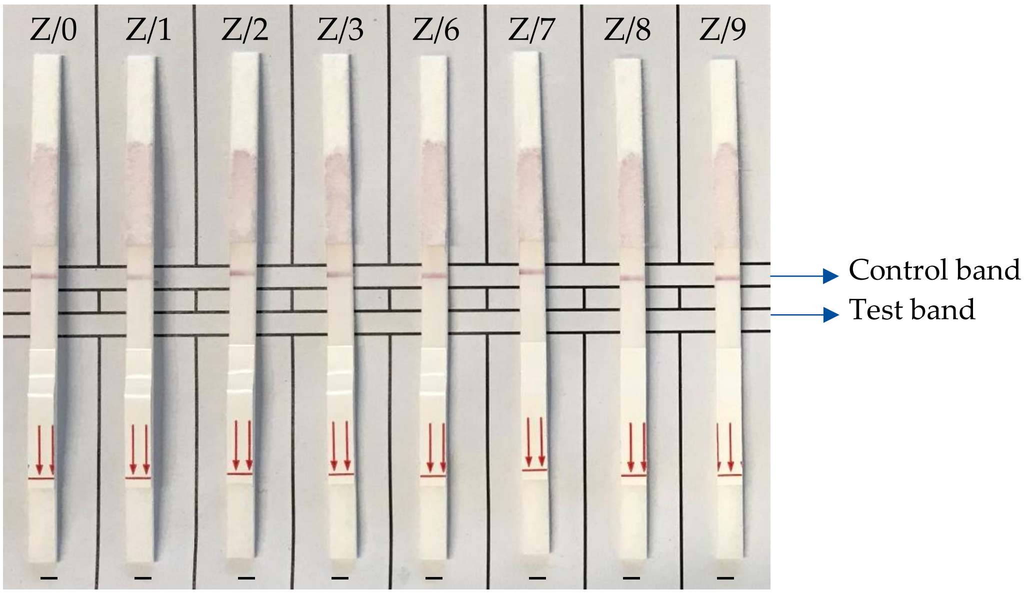

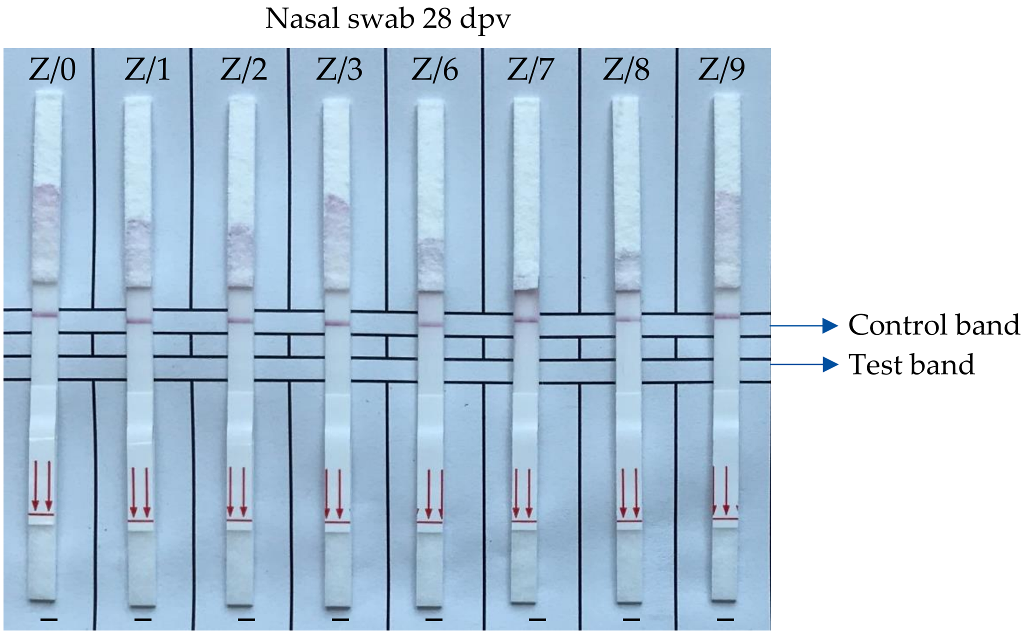
Nasal swab 28 dpv
Z/0
Z/1
Z/2
Z/3
Z/6
Z/7
Z/8
Z/9
Control band
Test band
–
–
–
–
–
–
–
–

Figure S1b. ID rapid PPR antigen dipstick field test results of conjunctival swab. Negative (-) control band visible test band not, questionable ((+)) control band visible and barely visible test band, low positive (+) test band visible but not strong color as control band, moderate positive (++) test band same visible color as control band, high positive (+++) test band stronger color than control band.

Conjunctival swab 0 dpv

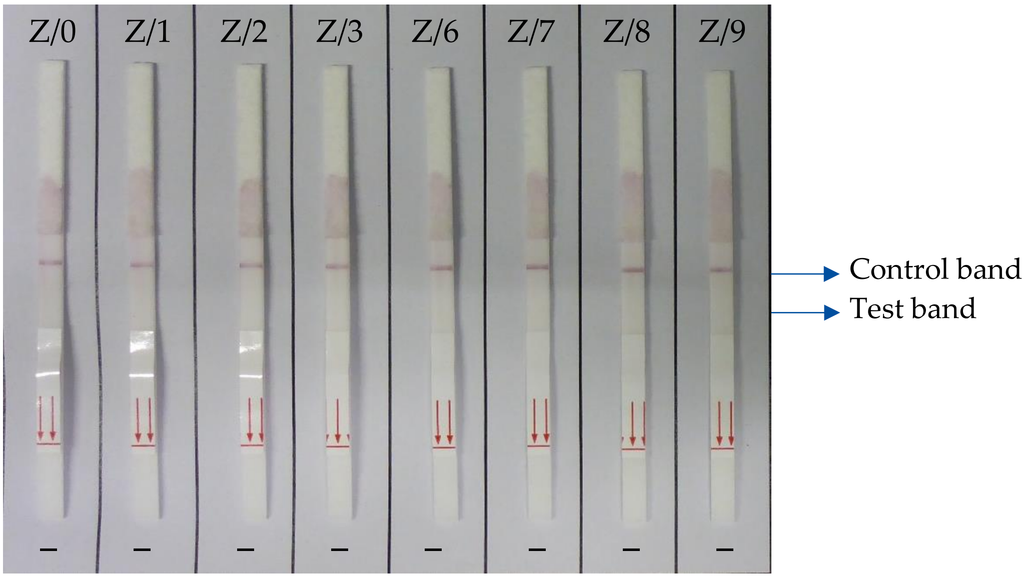

Conjunctival swab 3 dpv

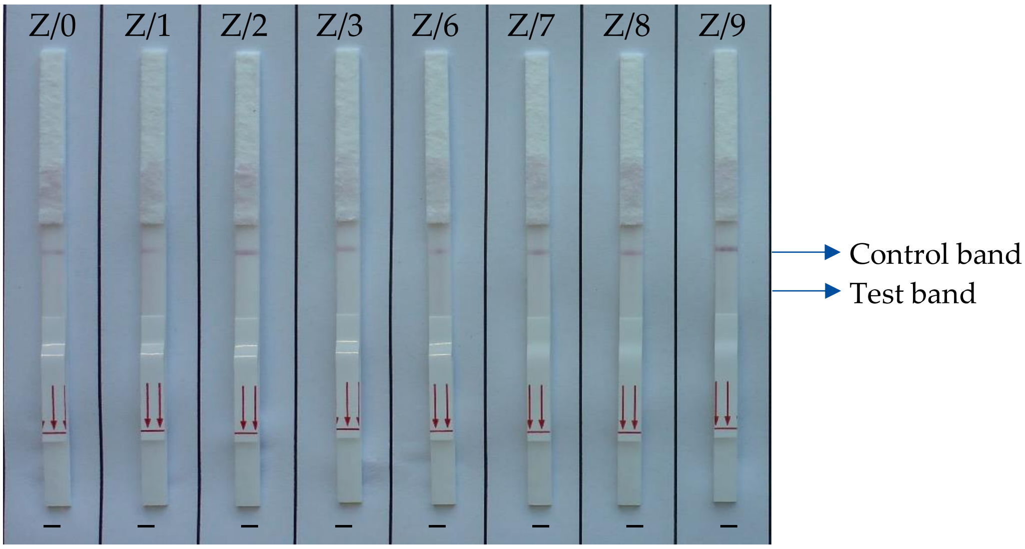

Conjunctival swab 5 dpv

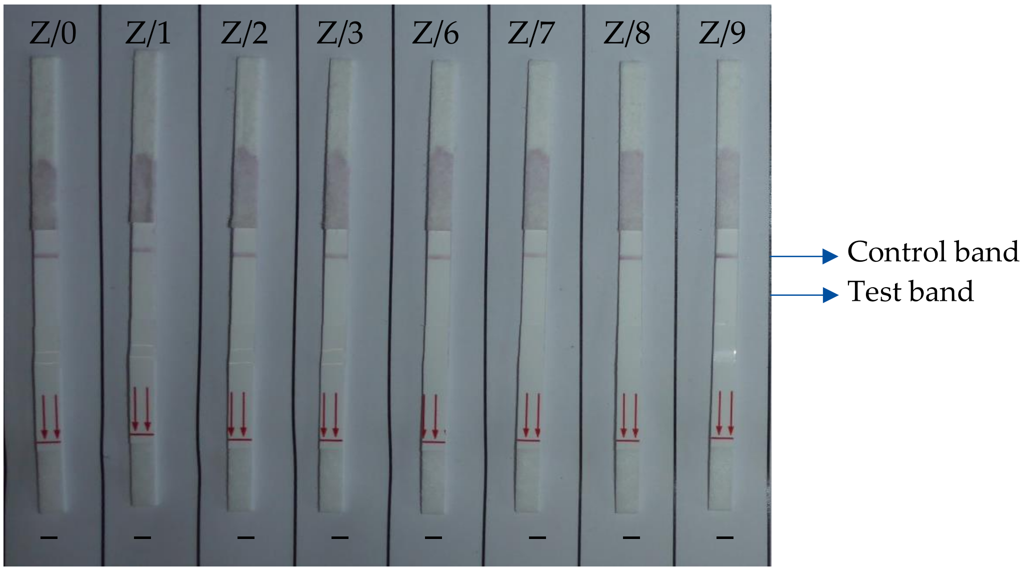

Conjunctival swab 7 dpv

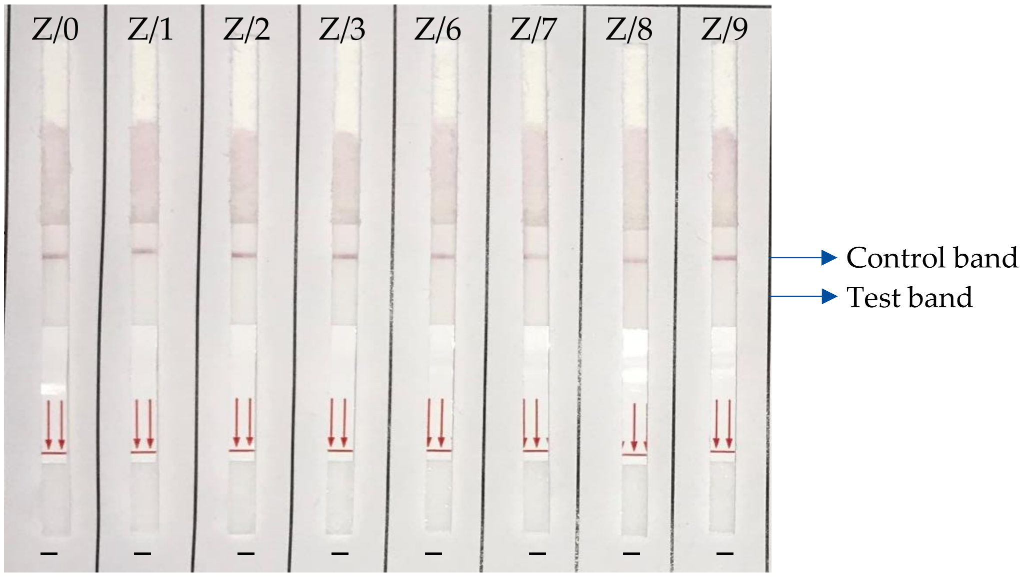

Conjunctival swab 10 dpv

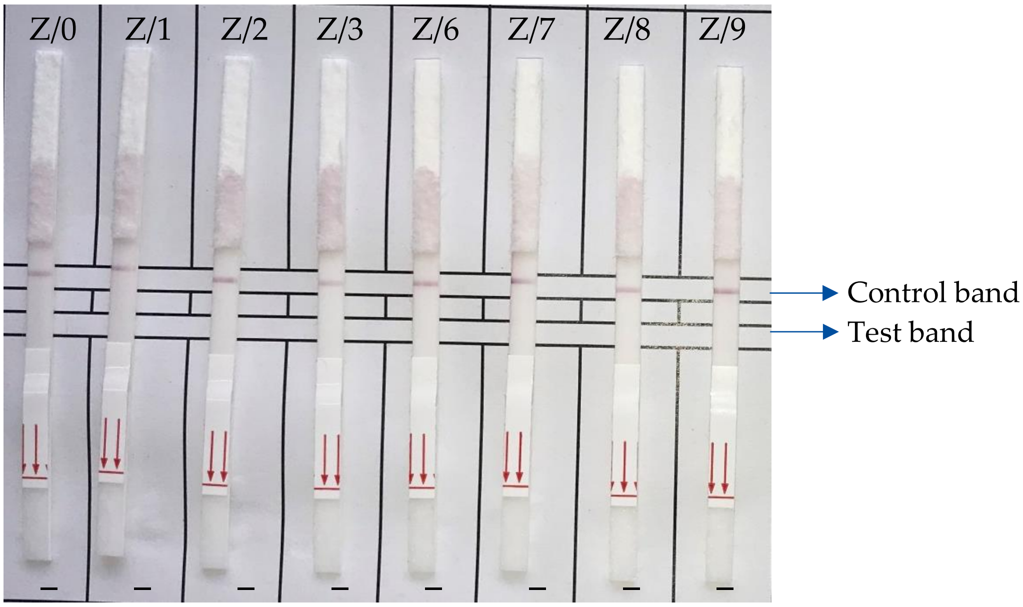

Conjunctival swab 12 dpv

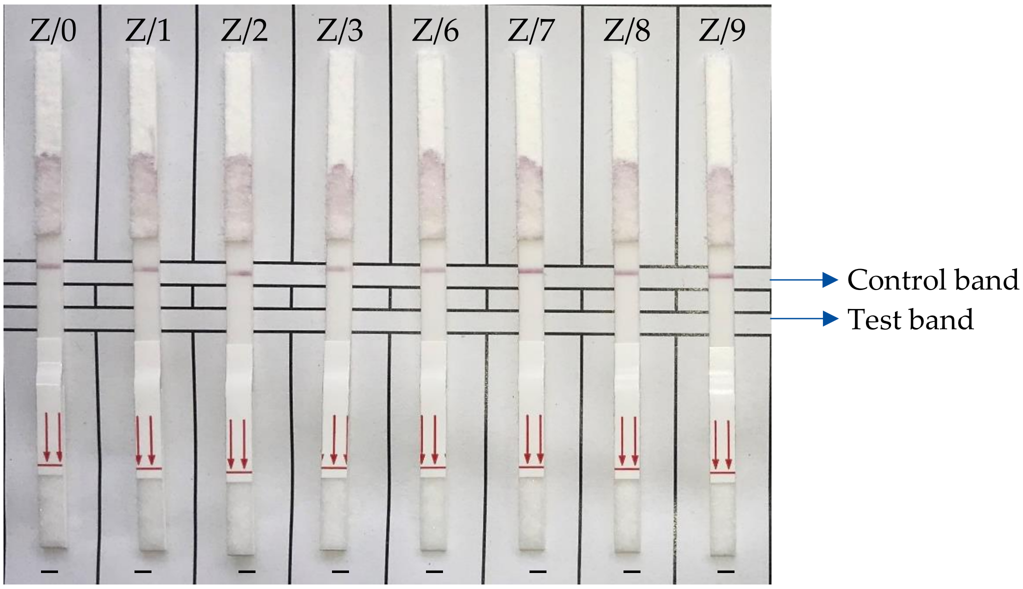

Conjunctival swab 14 dpv

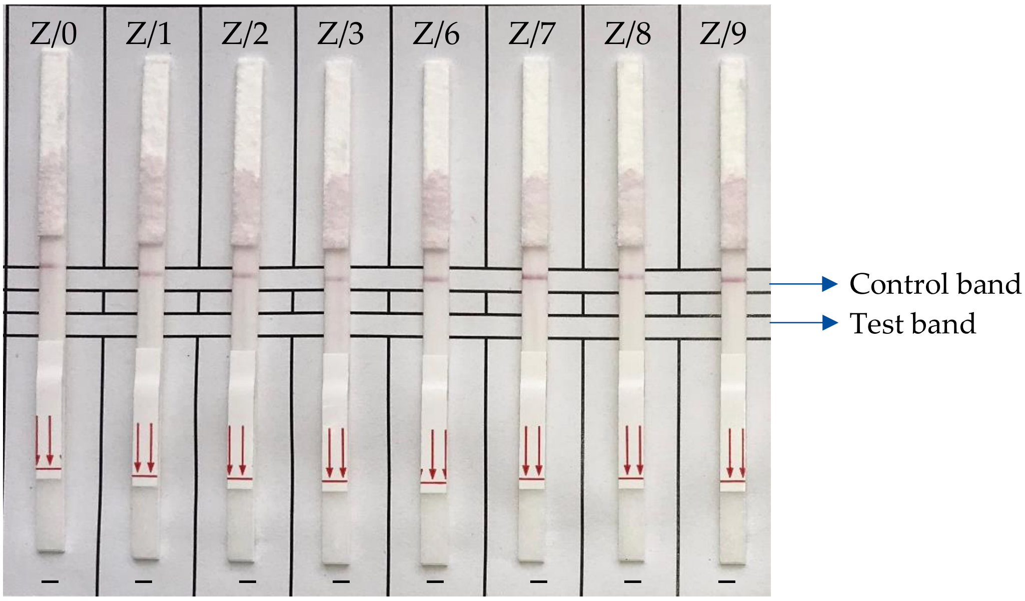

Conjunctival swab 17 dpv

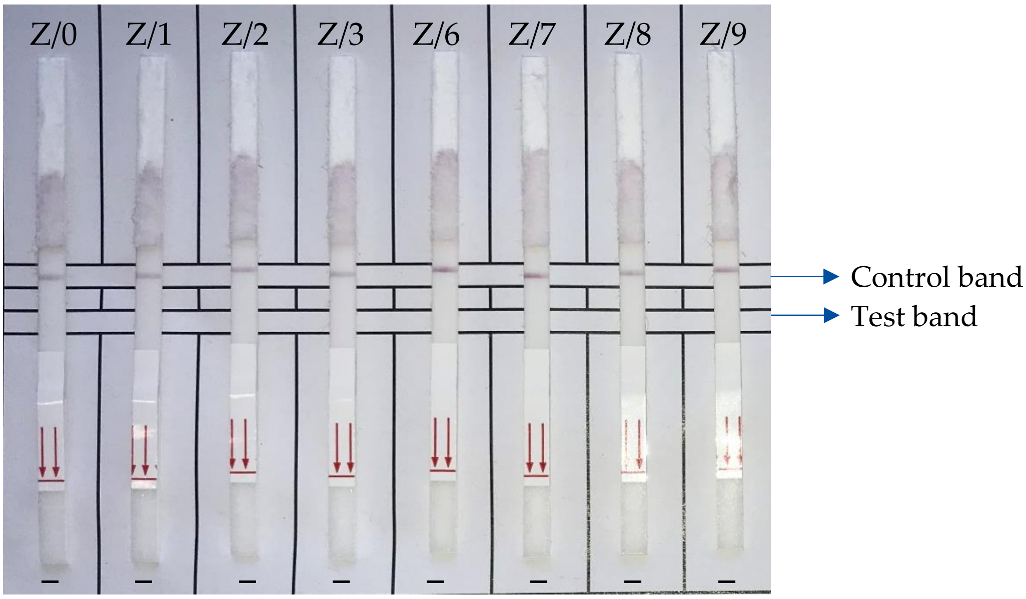

Conjunctival swab 21 dpv

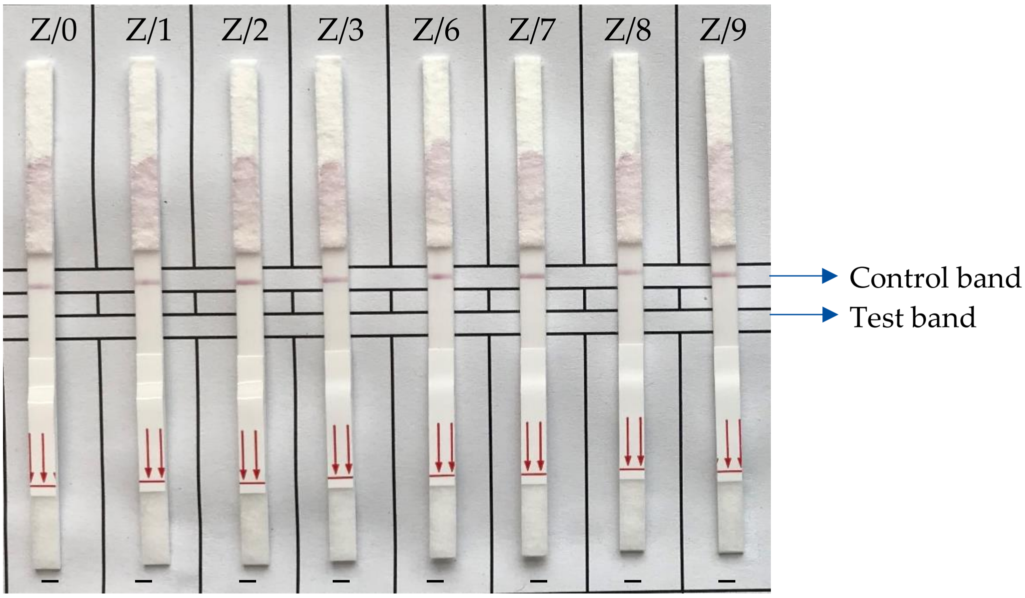

Conjunctival swab 24 dpv

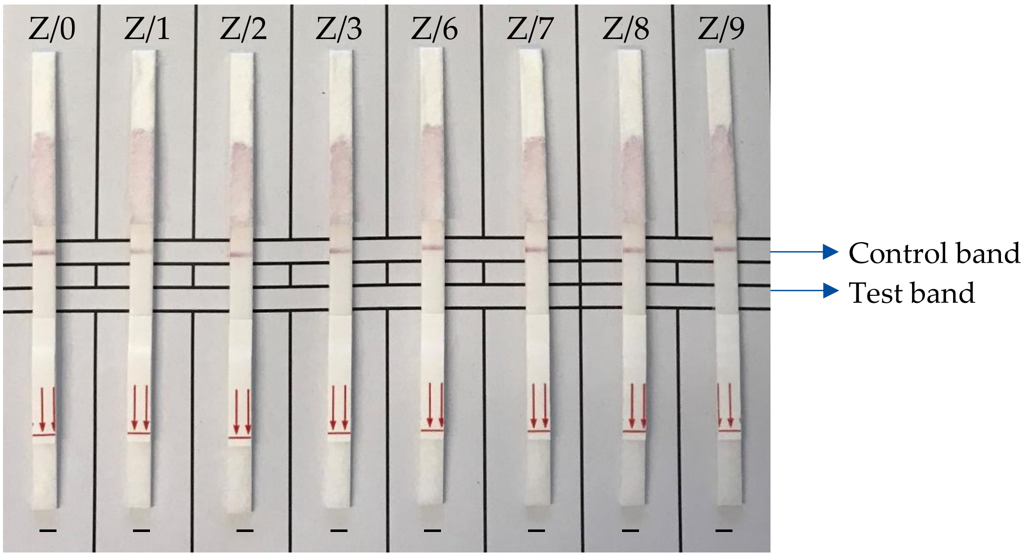

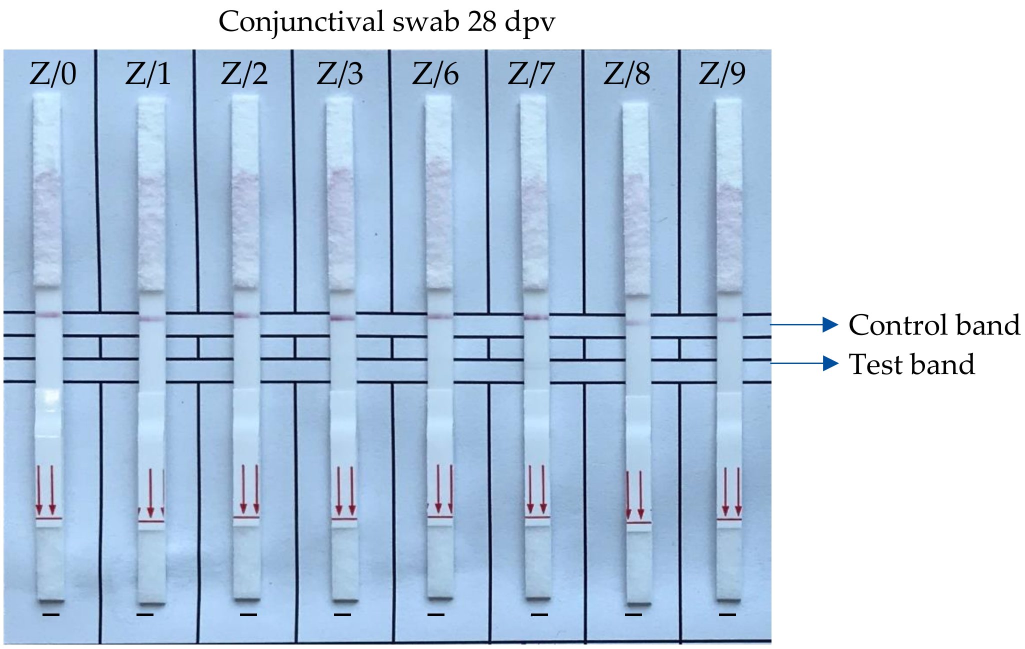
Conjunctival swab 28 dpv
Z/0
Z/1
Z/2
Z/3
Z/6
Z/7
Z/8
Z/9
Control band
Test band
